# Comparative Diagnostic Accuracy of Ultrasound, MRI, and Fine-Needle Aspiration Biopsy in the Preoperative Evaluation of Parotid Gland Tumors

**DOI:** 10.3390/jcm14041342

**Published:** 2025-02-18

**Authors:** Sebastian Stoia, Anca Ciurea, Mihaela Băciuț, Simion Bran, Gabriel Armencea, Emil Boțan, Manuela Lenghel, Tiberiu Tamaș, Rareș Mocan, Daniel Leucuța, Grigore Băciuț, Cristian Dinu

**Affiliations:** 1 Department of Oral and Maxillofacial Surgery and Radiology, Faculty of Dental Medicine, “Iuliu Hațieganu” University of Medicine and Pharmacy, 400012 Cluj-Napoca, Romania; 2 Department of Radiology, Faculty of Medicine, “Iuliu Hațieganu” University of Medicine and Pharmacy, 400012 Cluj-Napoca, Romania; 3 Department of Pathology, Emergency County Hospital, 600114 Cluj-Napoca, Romania; 4 Department of Medical Informatics and Biostatistics, “Iuliu Hațieganu” University of Medicine and Pharmacy, 400012 Cluj-Napoca, Romania

**Keywords:** parotid gland, tumors, diagnosis, magnetic resonance imaging, ultrasound, fine-needle aspiration biopsy

## Abstract

**Background:** The objective of this study was to compare the value of ultrasound (US), magnetic resonance imaging (MRI), and US-guided fine-needle aspiration biopsy (FNAB) in the preoperative evaluation of parotid tumors. **Methods:** A three-year prospective study, including 35 patients, was conducted. Preoperative ultrasound, MRI, and US-guided FNAB were performed on each patient, after which an imaging and cytological diagnosis was obtained. Each patient underwent surgical treatment. The imaging and cytological diagnoses were compared with the histopathological reports. **Results:** Ultrasound and MRI showed the same diagnostic performance in discriminating benign from malignant parotid tumors: sensitivity—80%, specificity—97%, and accuracy—94%. In this regard, FNAB registered a sensitivity, specificity, and accuracy of 100%, 97%, and 97%, respectively. US, MRI, and FNAB were recorded as having high diagnostic accuracy in the detection of pleomorphic adenoma and Warthin tumors. **Conclusions:** Ultrasound and US-guided FNAB allow for the preoperative differential diagnosis of parotid tumors located in the superficial lobe. When US and FNAB results are inconclusive, MRI becomes mandatory.

## 1. Introduction

Salivary gland tumors represent 3–6% of head and neck tumors. Among salivary gland tumors, benign histologies account for over 65%. The greater part of the salivary glands’ tumors (59–80%) occur in the parotid glands. The most common benign tumor is pleomorphic adenoma (60–70%), followed by Warthin tumor (17–30%). Mucoepidermoid carcinoma ranks first among malignant parotid tumors [1,2,3,4,5,6].

Surgery is the primary treatment for parotid gland tumors. At present, parotidectomy or extracapsular dissection are the most widely accepted surgical techniques for parotid gland tumors.

The surgical approach is planned differently based on the parotid tumor’s histological type and characteristics. Consequently, the selection of the ideal surgical treatment requires precise preoperative imaging and cytological diagnosis [7,8,9].

Salivary gland tumors present a wide histological variety, with the classification of the World Health Organization (2017) presenting 31 histological types [1]. This great histological diversity within parotid tumors, the absolute contraindication of open biopsies in the territory of the parotid gland, and the anatomical particularities of this complex region make the preoperative diagnosis of these tumors difficult and challenging for clinicians. As an alternative to open biopsy, fine-needle aspiration biopsy (FNAB) is commonly performed for parotid tumors [10,11]. FNAB has high specificity and sensitivity in differentiating neoplastic from non-neoplastic parotid gland pathology, as well as in distinguishing between benign and malignant parotid tumors [10,11,12,13,14]. The overlap of morphological features of parotid gland tumors may lead to inconclusive FNAB results [15,16].

In current clinical practice, US is often used as a primary imaging modality for diagnosing parotid tumors. Ultrasound allows for the differentiation of benign tumors from malignant ones with high accuracy and contributes to the guidance of FNAB, thus improving the accuracy of FNAB [17,18]. Ultrasound can differentiate pleomorphic adenoma from Warthin tumor with high values of sensitivity, specificity, and accuracy [19,20,21].

Multiparametric magnetic resonance imaging (especially diffusion-weighted imaging—DWI and dynamic contrast-enhanced sequences – DCE) allows for an accurate preoperative differential diagnosis of parotid tumors, with it being accepted at present as the gold standard imaging examination [9,22].

The diagnostic value and clinical utility of ultrasound, MRI, and fine-needle aspiration biopsy in the differential diagnosis of parotid tumors prior to surgery are currently intensely debated subjects in the literature.

This study describes the strengths and limitations of the diagnostic accuracy of US, MRI, and FNAB for the preoperative evaluation of parotid tumors. Moreover, this study aimed to conduct a comparison between the diagnostic performance of the two imaging investigations (US and MRI) and the cytological one (FNAB) used in this regard.

After we analyzed the literature on this topic, we found that no other studies published in the literature have compared the diagnostic performance of the three investigations (US, MRI, and FNAB).

Comparing the three investigations can provide clinicians with an overview of the diagnostic power of each investigation, as well as the limitations of these three investigations. In addition, it allows clinicians to use these investigations judiciously and at the same time to avoid additional investigations that are not necessary in specific clinical situations.

## 2. Materials and Methods

### 2.1. Study Design

A prospective, single-center study carried out over the course of 3 years (2019–2022) was conducted in the Department of Maxillofacial Surgery at the Emergency Clinical County Hospital in Cluj-Napoca, Romania. The ethics committees of the University of Medicine and Pharmacy “Iuliu Hațieganu” Cluj-Napoca, Romania (protocol number 480 from 21 November 2019), and the Emergency Clinical County Hospital, Cluj-Napoca, Romania (protocol number 36752 from 17 December 2019), both gave their approval for the present study.

### 2.2. Participants

Patients diagnosed with primary parotid tumors suitable for surgical excision were included in the study. The following exclusion criteria were applied: patients with recurrent parotid tumors, patients who did not consent to the surgical treatment, lack of surgical indication or general contraindication for surgery, patients with contraindications for FNAB or MRI, or patients with parotid tumors in whom ultrasonography was not the first chosen imaging method. In total, 35 patients met the study’s inclusion and exclusion criteria. All patients who participated in this study signed an informed consent form.

### 2.3. Data Collection

For each patient, an ultrasound was performed as the first imaging investigation, followed by a multiparametric MRI with diffusion-weighted imaging (DWI) and dynamic contrast sequences (DCEs). After these investigations, an imaging diagnostic protocol was established. The US and MRI examinations were performed by the same radiologist.

An FNAB of the parotid tumor was performed in each patient included in the study, after completion of US and MRI investigations. This procedure was performed by the same maxillofacial surgeon in all patients.

The type of surgical intervention was decided based on the imaging and cytological reports, with each patient undergoing surgery for excision of the parotid gland tumor. The preoperative ultrasound, MRI, and cytological diagnoses were compared with the final histopathological report following the surgical excision of the parotid tumor.

US and MRI examinations were performed and analyzed by a single radiologist with over 15 years of experience in this field. The Signa Explorer 1.5 Tesla, General Electric (GE) system with 16 dedicated channels was used for the MRI examination. The ultrasound was performed using a Hitachi EUB 8500 equipment with a linear transducer using a variable frequency between 6.5 and 13 MHz.

For DCE-MRI, a baseline T1 mapping before administration of contrast medium was the first step, followed by dynamic data acquisition with 40 phases performed after gadolinium injection. The temporal resolution was 4 seconds. An axial 3D T1 DISCO sequence was used for the acquisition of DCE-MRI images. The parameters used were TR (repetition time)—4 ms and TE (time echo)—1.5 ms. For DWI sequences, two b-values were used: 0 and 1000 s/mm^2^.

All of the fine-needle aspiration biopsies were performed using a 22-gauge (G) needle of 6–8 cm length attached to a 10 ml syringe fixed in a “pistol grip” syringe holder produced by “Medax Sonogun”. The harvested material was expressed on a microscopic slide and examined on the same day. All of the fine-needle aspiration biopsies were performed by a single maxillofacial surgeon and were guided with ultrasound by the same radiologist who previously performed the ultrasound and MRI examinations of the parotid tumors.

A single pathologist with more than 15 years of experience in this field conducted all of the cytological analyses of the harvested samples.

During the study period, the same equipment, protocols, and techniques were used by the same operators.

All data were collected by the study director and stored in electronic format (Microsoft Excel).

**Figure 1:**
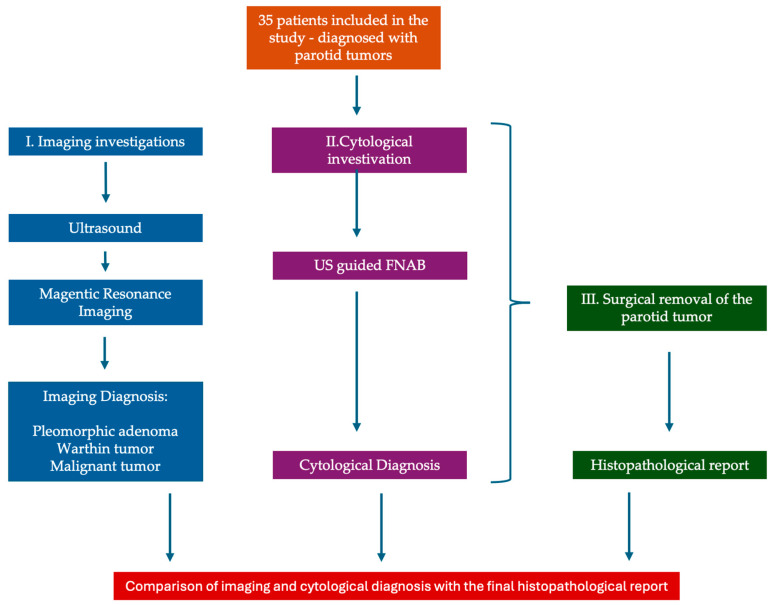
Data collection and study protocol—flowchart.

The data collection and study protocol are presented in Figure 1 as a flowchart.

### 2.4. Statistical Methods

Regarding the statistical analysis absolute frequencies and percentages were used to describe categorical data. Quantitative data were reported as medians and interquartile ranges. When applicable, the Chi-squared test or Fisher’s exact test was utilized for comparing two independent groups of categorical data, and for skewed quantitative data, the Wilcoxon rank-sum test was applied. To optimize the Youden index, the ideal cut-off point was determined, and the sensitivity and specificity were computed. A contingency table was used to assess the diagnostic accuracy of ultrasound, MRI, and FNAB as index tests in comparison to the histopathological report. Sensitivity, specificity, positive and negative predictive values, positive and negative likelihood ratios, diagnostic odds ratios, Youden index, and accuracy were then calculated, along with 95% confidence intervals. The statistically significant value was determined to be 0.05, and the two-tailed *p*-value was computed for each statistical test. Pairwise comparisons between the three diagnostic methods (ultrasound, MRI, and FNAB) were tested with the exact Mc Nemar test. By having three paired comparisons between the diagnostic methods, to prevent alpha inflation (the probability of coming to at least one false conclusion in a series of hypothesis tests), a value of 0.05 divided by three (0.0167) was considered statistically significant. When also considering subgroup comparisons for benign adenoma and Warthin tumor, a *p*-value of 0.05 divided by nine (0.0056) can be considered statistically significant. All statistical analyses were conducted using R version 4.1.2. (R Foundation for Statistical Computing, Vienna, Austria) [23].

## 3. Results

### 3.1. Patient Demographic Data and Parotid Tumor Characteristics

A total number of 35 patients diagnosed with primary parotid tumors were included in this study. Of these patients, 30 presented with benign tumors and 5 were diagnosed with malignant tumors. Among these patients, 63% were women and 37% were men. The median age was 56 years, and the age range was between 21 and 71 years. The patient’s demographic data and the parotid tumor characteristics are presented in detail in Table 1.

The univariate statistical analysis showed statistical differences between benign and malignant parotid tumor characteristics in terms of patient age, living environment, and tumor location within the parotid gland. The mean age for patients with malignant parotid tumors was 68 years and 52 years for patients with benign tumors.

Most of the malignant parotid tumors presented extension in the deep parotid lobe (80%).

Patients with benign parotid tumors had a mean age of 52 years, and those with malignant parotid tumors had a mean age of 68 years. Regarding gender, for the benign tumor group, 20 patients (67%) were female, and for the malignant tumor group, 2 patients were male (40%). In total, 26 (87%) patients with benign parotid tumors came from the urban area, and 2 patients (40%) with malignant tumors originated from the urban area. In the benign tumor group, 16 tumors (53%) were located in the left parotid gland, and 26 (87%) were located in the superficial lobe. In the malignant parotid tumor group, 1 tumor (20%) was located in the left parotid gland, and 1 tumor (20%) was located in the superficial lobe.

### 3.2. Distribution of Histopathologic Subtypes of Parotid Gland Tumors

The final histopathologic diagnosis was established after the surgical removal of the primary parotid tumor. This study included 35 parotid tumors, of which 86% were benign tumors and 14% were malignant. The histopathologic subtype distribution of the parotid gland tumors included in this study is shown in detail in Table 2.

### 3.3. Ultrasound, MRI, and FNAB Evaluation—Differential Diagnosis Performance Between Benign and Malignant Parotid Tumors

Using magnetic resonance imaging (MRI), for the differential diagnosis between benign and malignant parotid tumors, the same diagnostic performance as with ultrasound was obtained.

The diagnostic performance of fine-needle aspiration biopsy (FNAB) in the differential diagnosis of benign parotid tumors from malignant tumors was analyzed by comparing the preoperative FNAB diagnosis with the final postoperative histopathological report.

A detailed comparison of the diagnostic performance of the two imaging (US and MRI) and cytological (FNAB) methods utilized to distinguish between benign and malignant parotid tumors can be found in Table 3.

Furthermore, comparisons between the diagnostic performane of US, MRI, and FNAB were assessed. Each of the diagnostic methods were compared, and the *p*-value for the Mc Nemar exact test was 1, for all pairs. Using the significance levels of 0.0167 or 0.0056 for multiple comparisons, a statistically significant difference cannot be stated.

### 3.4. US, MRI, and FNAB Evaluation—Diagnostic Performance in Detecting Pleomorphic Adenoma and Warthin Tumor

In this study, we analyzed the ability of US, MRI, and FNAB to diagnose pleomorphic adenoma among parotid tumors. Diagnostic performance in this sense of the three methods used, namely the imaging methods (US and MRI) and the cytological method (FNAB), is presented comparatively and in detail in Table 4. Ultrasound and MRI demonstrated the same diagnostic performance.

Comparisons of the diagnostic performance of US, MRI, and FNAB in detecting pleomorphic adenoma were evaluated. The *p*-value for the exact Mc Nemar test for all three pairwise comparisons was 1, for all pairs. Using the significance level of 0.0167 or 0.0056 for multiple comparisons, a statistically significant difference cannot be stated.

US, MRI, and FNAB were evaluated for their ability to diagnose Warthin tumors among other parotid gland tumors. In Table 5, the diagnostic performance of the three adopted procedures, namely the imaging examinations (US and MRI) and the cytological technique (FNAB), is compared and described in detail. When used to identify Warthin tumors, ultrasound and MRI showed the same diagnostic performance.

We compared the diagnostic performance of US, MRI, and FNAB in detecting Warthin tumors. The *p*-value for the exact Mc Nemar test for all pairwise comparisons was 1. Using the significance level of 0.0167 or 0.0056 for multiple comparisons, a statistically significant difference cannot be stated.

We performed a detailed evaluation of histopathological entities misdiagnosed when using the three diagnostic tools used by us in this study (US, MRI, and FNAB). Following this analysis, we obtained a series of relevant data that will be presented below.

Therefore, the ultrasound examination provided diagnostic errors in the case of 3 pleomorphic adenomas from the total of 12 tumors included in the study: 1 tumor was misdiagnosed as malignant, 1 was misdiagnosed as a Warthin tumor, and in another case, the histological subtype could not be specified, with it being classified as a benign tumor. In addition, ultrasound confused mucoepidermoid carcinoma with pleomorphic adenoma, and 1 tumor among the 16 Warthin tumors was confused with pleomorphic adenoma.

The same diagnostic errors and confusion were encountered when magnetic resonance imaging was used.

Regarding the FNAB technique, diagnostic errors occurred in the case of 1 tumor out of the 12 pleomorphic adenomas, with this tumor being misdiagnosed as a Warthin tumor. The mucoepidermoid carcinoma and the mucous retention cyst were misdiagnosed as Warthin tumors and two Warthin tumors were misdiagnosed as pleomorphic adenomas when using the FNAB technique.

## 4. Discussion

In terms of age, urban or rural origin of the patients, and tumor location inside the parotid gland, the study’s findings showed differences between benign and malignant parotid tumors. In line with the findings of other studies that have been reported in the literature, patients with malignant tumors had a higher mean age than patients with benign tumors (68 versus 52 years) [3,6,9].

A high percentage (87%) of patients with benign parotid tumors resided in an urban environment. Regarding the location of tumors within the parotid gland, the majority of benign parotid tumors were located in the superficial lobe (87%), in accordance with the findings of other studies [9,19,24].

Our study results showed that Warthin tumors comprised 53% of benign parotid tumors, followed by pleomorphic adenomas (40%) in second place. This result contradicts most previously published studies [1,3,4,6]. Nonetheless, Psychogios et al. reported findings similar to ours, indicating a 42% incidence of Warthin tumors [25].

In accordance with the literature, our study confirmed the great histological diversity of parotid gland tumors by identifying nine different histological types among the 35 parotid tumors investigated within the present study [1,3,4,6,9].

**Figure 2:**
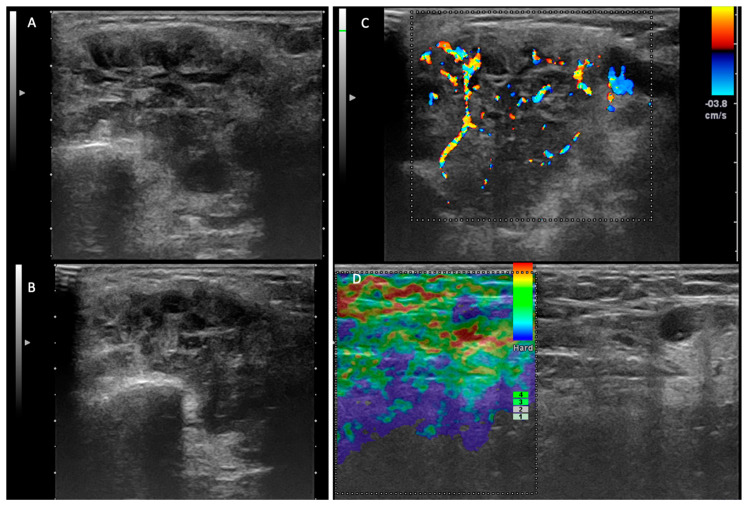
Malignant tumor—left parotid gland. (**A**,**B**) Two-dimensional ultrasound that reveals an inhomogeneous tumor, with areas of necrosis and irregular and infiltrative contour. (**C**) The color Doppler examination demonstrates an irregular, anarchic distribution of the vascular signal. (**D**) Elastography examination—laterocervical adenopathy with areas of intranodal stiffness.

The differentiation of a low-grade malignant parotid tumor from a benign tumor is the first impediment when using ultrasound examination. In accordance with the US criteria of malignancy, recognized in the literature, we assumed that the irregular shape, poorly defined margins, heterogeneous and hypoechoic structure, high and anarchic vascularization, and the presence of loco-regional lymphadenopathies are probable US criteria of malignancy (Figure 2) [17,19,26].

Because of the overlap of the ADC value between low-grade malignant parotid tumors and Warthin tumors, this impediment can occur even when multiparametric MRI is utilized [27]. In addition, the literature reports that Warthin tumors, parotid lymphomas, and other low-grade malignant parotid tumors may register overlaps of the C-type TIC curve or ADC values when DCE-MRI or DWI-MRI sequences are used [9,28].

This study examined and compared the diagnostic potential of US and MRI in discriminating benign from malignant parotid tumors. The detailed results of this analysis are presented in Table 3. The preoperative ultrasound examination and the MRI investigation demonstrated the same high values of sensitivity, specificity, and accuracy in distinguishing benign from malignant parotid tumors. Using ultrasound for the differential diagnosis of benign and malignant parotid tumors, in their study, Rzepakowska et al. (2017) obtained US diagnostic performance comparable to our results, reporting the following values of sensitivity, specificity, and accuracy: 60%, 95%, and 90%, respectively [19]. In contrast, according to a recent literature review, the diagnostic performance of ultrasound in discriminating benign from malignant parotid tumors was lower, with reported accuracy values ranging from 62 to 65%, sensitivity values ranging from 39 to 88%, and specificity values ranging from 67 to 90% [17].

The relatively large differences in ultrasound diagnostic performance in differentiating benign from malignant parotid tumors reported in the literature can be explained by the fact that this examination is operator-dependent and has some limitations in properly assessing parotid tumors located in the deep lobe of the gland. The presence of the mandibular bone structure limits US examination in the case of parotid tumors located posterior to it or in the case of tumors located in the deep parotid lobe or with extension to the parapharyngeal space.

Regarding the use of MRI for the differentiation of benign parotid tumors from malignant ones, comparable results in terms of sensitivity, specificity, and accuracy have been obtained in other studies [11,27]. In a recent study, focused on MRI investigation, we reported a sensibility of 82%, a specificity of 89%, and an accuracy of 92% for the imaging investigation used in this sense [9]. Elmokadem et al. (2018) reported a sensitivity of 95%, a specificity of 97%, and an accuracy of 97% when using DCE-MRI in differentiating benign from malignant parotid tumors [28].

**Figure 3:**
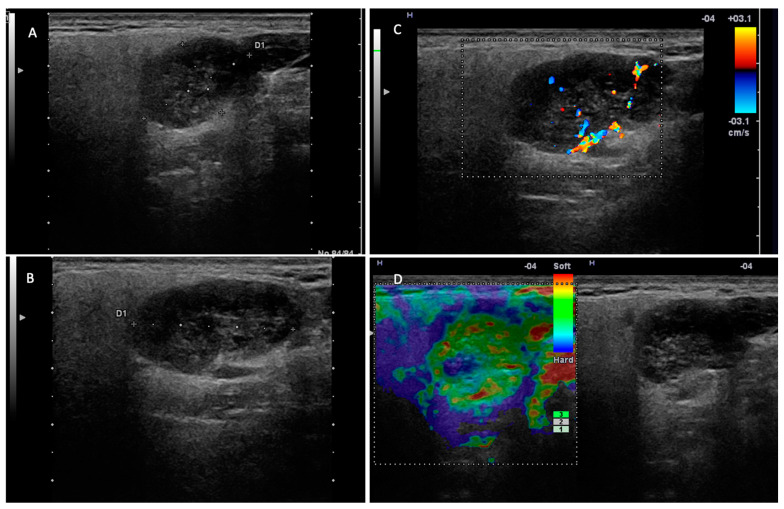
Warthin tumor—right parotid gland. (**A**,**B**) Two-dimensional examination revealing a hypoechoic, inhomogeneous nodule with areas of intralesional fluid degeneration, with a slightly irregular contour but well demarcated from the adjacent parotid parenchyma. (**C**) The color Doppler examination reveals intratumoral vascularization. (**D**) Elastography examination with peripheral stiffness and intralesional soft appearance.

**Figure 4:**
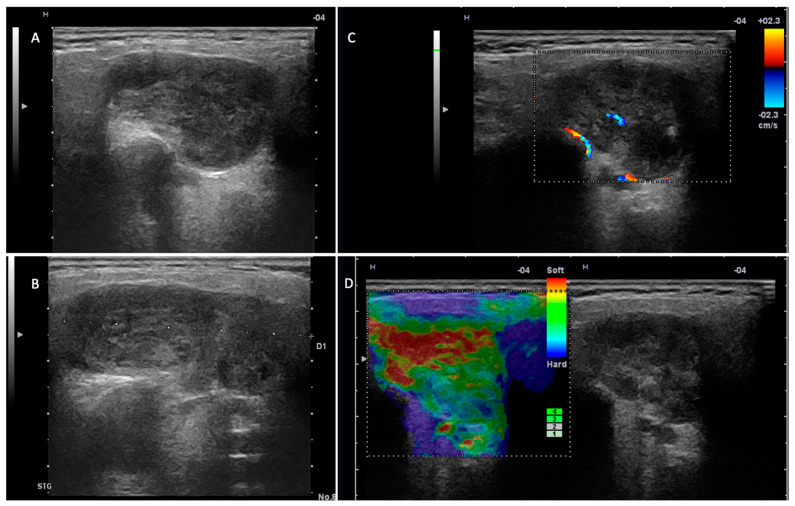
Pleomorphic adenoma—left parotid gland. (**A**,**B**) Two-dimensional examination revealing a hypoechoic nodule, with an irregular contour, slightly lobulated, and well demarcated from the adjacent parotid parenchyma. (**C**) The color Doppler examination reveals a reduced intralesional vascular signal. (**D**) Elastography examination with a predominantly soft intratumoral appearance.

In this study, we investigated the diagnostic performance of ultrasound and MRI in the differentiation of pleomorphic adenomas and Warthin tumors from other parotid tumors. Among the most important ultrasound parameters that contributed to the US diagnosis of the two tumor entities were as follows: the tumor vascular pattern, the acoustic enhancement, the density and composition of the tumor content, and the presence of cystic areas (Figure 3 and Figure 4).

According to Mansour et al. (2012), the tumor vascular pattern and acoustic enhancement are the most important factors in distinguishing pleomorphic adenomas from Warthin tumors, when using ultrasound examination [26]. Matsuda et al. (2017) implemented a novel system to classify tumor composition, utilizing anechoic areas as a substitute for homogeneity differences in parotid tumors, thereby distinguishing pleomorphic adenomas from Warthin tumors [29].

**Figure 5:**
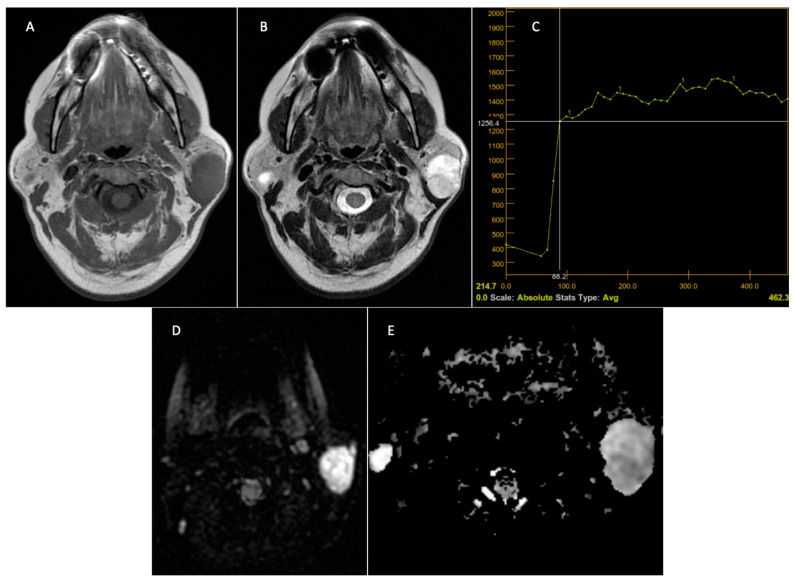
Pleomorphic adenoma—left parotid gland. Preoperative MRI. T1 sequence with a hypointense signal (**A**). T2 sequence with a discrete inhomogeneous hyperintense signal (**B**). DCE-MRI—slow and progressive contrast uptake with late washout—TIC curve type A, typical appearance of pleomorphic adenoma **(C)**. The DWI—MRI sequences (**D**) demonstrate T2 appearance, “shine through”, with a hypersignal on the DWI sequence and ADC, with the ADC map (**E**) having a value of 1.6 × 10^–3^ mm^2^/s.

**Figure 6:**
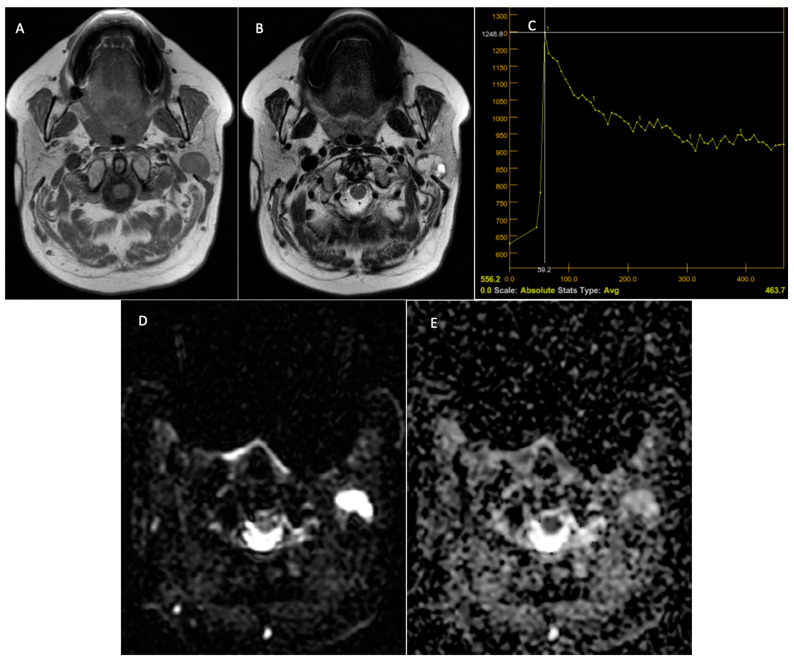
Warthin tumor—left parotid tumor. Preoperative MRI. T1-weighted sequence with a hypointense signal at the level of the tumor, a well-demarcated lesion (**A**). T2 sequence—mixed signal at the level of the lesion and the presence of cystic areas at the level of the lesion (**B**). DCE-MRI—C-type TIC curve, with rapid contrast uptake and rapid washout (**C**). The DWI-MRI sequences (**D**) demonstrate intratumoral diffusion restriction, with the ADC map (**E**) having a value of 0.73 × 10^−3^ mm^2^/s.

**Figure 7:**
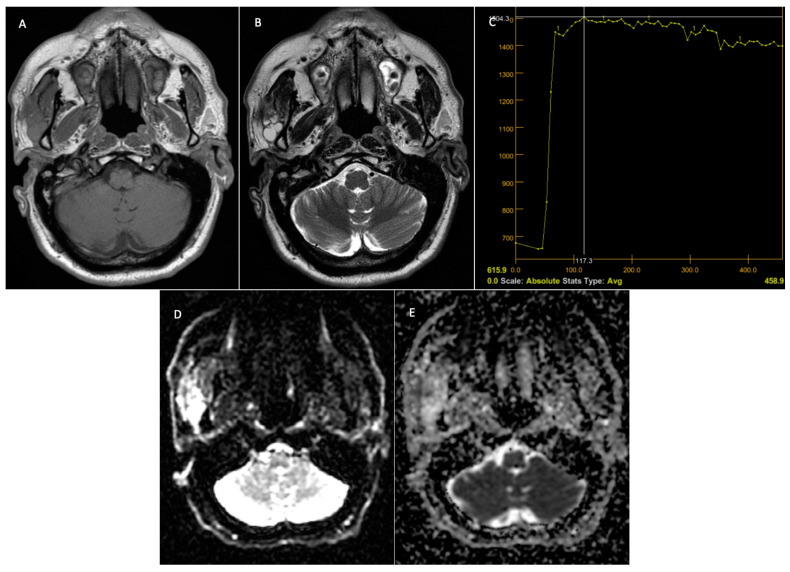
Malignant tumor—right parotid gland. Preoperative MRI. T1 sequence with a hypointense signal in the tumor (**A**). T2 sequence—inhomogeneous signal at the level of the lesion and the presence of micro-calcifications at the level of the lesion (**B**). DCE—MRI—TIC curve type B, with progressive contrast uptake followed by a plateau phase and washout (**C**). The DWI—MRI sequences (**D**) demonstrate restriction at the level of the solid component of the tumor, with the ADC map (**E**) having a value of 1 × 10^−3^ mm^2^/s.

Regarding the multiparametric MRI investigation, the most important parameters used to discriminate pleomorphic adenoma and Warthin tumor from other parotid tumors were as follows: T1-weighted image sequences, T2-weighted image sequences, time–intensity curves (TIC), apparent diffusion coefficient (ADC), and time to peak (TTP) (Figure 5, Figure 6 and Figure 7).

In the present study, ultrasound and MRI demonstrated high diagnostic performance in detecting pleomorphic adenomas and Warthin tumors among other parotid tumors, reporting the same sensitivity, specificity, and accuracy for pleomorphic adenoma and Warthin tumors.

Biaek et al. (2003) reported a sensitivity of 64%, a specificity of 91%, and an accuracy of 77% in the detection of pleomorphic adenoma among other parotid tumor entities using ultrasound examination based on criteria of polycyclic form, distinct margins, and structure without cystic areas [30].

Rzepakowska et al. (2017) researched in their study the ability of US to differentiate between pleomorphic adenoma and Warthin tumors, reporting a sensitivity of 62%, a specificity of 82%, and an accuracy of 73% [19].

Lanišnik et al. (2020) reported in their study a final accuracy for ultrasound in the diagnosis of malignant parotid tumors, pleomorphic adenoma, and Warthin tumors of 91%, 81%, and 77% respectively [31].

Multiparametric MRI has been utilized by multiple investigators to accurately distinguish pleomorphic adenomas from Warthin tumors, demonstrating relatively high sensitivity, specificity, and accuracy [9,11,27]. In a recent study using multiparametric MRI for parotid tumor diagnosis, we demonstrated a sensitivity of 94%, a specificity of 100%, and an accuracy of 98% for MRI in distinguishing pleomorphic adenoma from Warthin tumors [9]. Elmokadem et al. (2018) concluded in their study that DWI-MRI discriminated benign parotid tumors from malignant ones with a sensitivity of 91%, a specificity of 75%, and an accuracy of 85% [28]. For discriminating pleomorphic adenoma from malignant tumors, with DWI-MRI, the same authors reported a sensitivity of 93%, a specificity of 100%, and an accuracy of 97% [28].

In our study, most of the tumors were in the superficial parotid lobe (27 out of 35), with them being analyzed by an experienced radiologist specialized in head and neck pathology. All of this contributed to obtaining results that support the high performance of ultrasound in the differential diagnosis of benign parotid tumors from malignant ones and in detecting pleomorphic adenomas or Warthin tumors among other parotid tumor types.

In the present study, ultrasound and MRI provided misdiagnoses in five cases as follows:

- Three pleomorphic adenomas were confused with a malignant tumor, a Warthin tumor, and, in another case, the histological subtype could not be specified, but in this last case, the histopathological result proved to be a benign tumor as predicted by US and MRI (three false negative results).
- One mucoepidermoid carcinoma was confused with a pleomorphic adenoma (false negative result)
- One Warthin tumor was confused with a pleomorphic adenoma (false negative).

Rzepakowska et al. (2017) reported in their study, for the ultrasound diagnosis of pleomorphic adenoma, 10 false negative results and 5 false positive results [19]. Although considered a superior investigation, in these cases, MRI could not provide additional data that could have led to a more precise preoperative diagnosis. These results confirm the data from the literature that claim that even with the help of multiparametric MRI, there is a risk of misdiagnosis in the differentiation of parotid tumors because of the overlapping of imaging characteristics between benign tumors and malignant ones [28,32].

Currently, it is known that ultrasound has a reduced sensitivity in the differential diagnosis of parotid tumors as a result of the overlap of ultrasound characteristics between benign and low-grade malignant tumors. This fact is reported in the literature by numerous authors, with this also being encountered in our study through the confusion of the ultrasound diagnosis between a low-grade mucoepidermoid carcinoma and a pleomorphic adenoma [17,20,33,34,35].

In all misdiagnosed cases, the tumor size was less than 26 mm (ranging between 13 and 25 mm). The very small tumor dimension could be an explanation for the US diagnostic error, a fact also debated by other authors [19]. The small size of the tumor and the deep location of the tumor within the parotid gland may lead to difficulties in assessing all tumor characteristics during US examination. Well-defined margins and non-infiltrative margins are important diagnostic criteria for benign tumors, but often a low-grade malignant tumor, in its early stages, can present well-defined and non-infiltrative margins, with them being sources of confusion between malignant and benign parotid tumors, both on ultrasound and MRI evaluation. Another explanation for the confusion between malignant and benign tumors when using US or MRI could be the coexistence of inflammatory conditions in the parotid gland that can lead to an inhomogeneous aspect of the tumor and a false increase in the tumor vascular pattern, which are considered strong criteria for malignancy. The variable degrees of vascularity and cellularity of the parotid tumors may lead to an overlap between the TIC curves and ADC value between benign parotid tumors and malignant ones. The atypical aspect of the tumor on the MRI and US could lead to misdiagnosis. The absence of cystic areas would be an explanation for the confusion between a Warthin tumor and pleomorphic adenoma.

In this study, we analyzed in detail the diagnostic performance of the FNAB technique regarding the differentiation of benign parotid tumors from malignant ones, as well as the diagnostic value of this investigation in the detection of pleomorphic adenoma and Warthin tumors. The results are presented in detail and compared to those obtained with the help of ultrasound in Table 3, Table 4 and Table 5. In discriminating benign tumors from malignant ones, FNAB demonstrated a much higher sensitivity (100% vs. 80%), a similar specificity (97%), and a slightly higher accuracy (97% vs. 94%). In the diagnosis of pleomorphic adenoma, FNAB had a higher sensitivity (79% vs. 75%), a higher specificity (95% vs. 87%), and an increased accuracy (89% vs. 83%). In contrast, FNAB showed lower sensitivity, specificity, and accuracy compared to ultrasound when it was used in the diagnosis of Warthin tumors (82% vs. 88%, 89% vs. 94%, and 86% vs. 91%, respectively).

Data from the literature show that in differentiating benign parotid tumors from malignant ones, FNAB shows variable values of sensitivity (56–94%), specificity (75–98%), accuracy (61–98%), positive predictive value (60–90%), and negative predictive value (86–98%) [11]. Harb et al. (2020) reported a sensitivity of 79%, a specificity of 92%, a positive predictive value of 71%, and a negative predictive value of 95% for the FNAB technique used to differentiate benign from malignant parotid tumors [36]. In a similar study, Hamour et al. (2021) reported the following values for FNAB in the diagnosis of malignant parotid tumors: sensitivity—71%, specificity—99%, positive predictive value—83%, and negative predictive value—98% [37]. Dhanani et al. (2020) published results comparable to ours regarding the diagnostic performance of FNAB in the diagnosis of parotid tumors, with the following sensitivity, specificity, and accuracy values of the technique: 89%, 98%, and 93%, respectively [38]. Laninik et al. published a study in 2021 comparable to ours, obtaining the following sensitivity, specificity, and accuracy values for the ultrasound-guided FNAB technique: for the diagnosis of parotid gland malignant tumors: sensitivity—97%, specificity—55%, and accuracy—91%; for the diagnosis of pleomorphic adenoma: sensitivity—52%, specificity—95%, and accuracy—81%; and for the diagnosis of Warthin tumors: sensitivity—46%, specificity—98%, and accuracy—77% [31].

The variation in the diagnostic performance of FNAB may be attributed to technical errors, the experience of the clinician who performs the FNAB, the experience of the pathologist who interprets the sample, and the interval between sample collection and cytological analysis. In addition, the literature recognizes that ultrasound guidance of FNAB can substantially improve this technique’s diagnostic performance [11,18,31]. The high diagnostic performance of FNAB, supported by the high values of sensitivity, specificity, and accuracy obtained in this study, can be attributed to the fact that, in each case, the FNAB technique was guided by ultrasound and the samples were immediately examined by a pathologist with experience in head and neck tumors. Moreover, the FNAB exam is based on direct cellular analysis which can lead to a more accurate diagnosis.

According to the findings of our study, the FNAB approach was more effective than ultrasound in identifying pleomorphic adenoma and distinguishing benign from malignant parotid tumors. On the other hand, the FNAB approach demonstrated diagnostic performance inferior to ultrasound in the case of the diagnosis of Warthin tumors. This feature can be explained by the technical challenges associated with puncture due to the liquid content that is usually present in cases of this histological type. It may be difficult to express the harvested material on the microscope slide if the liquid content is aspirated during FNAB. Moreover, the fluid content of Warthin tumors could lead to reduced cellular loading of the needle during the back-and-forth movements required during the FNAB technique. Thus, when performed concurrently, the two investigations (US and FNAB) can complement one another and contribute to improving the precision of the final diagnosis.

**Figure 8:**
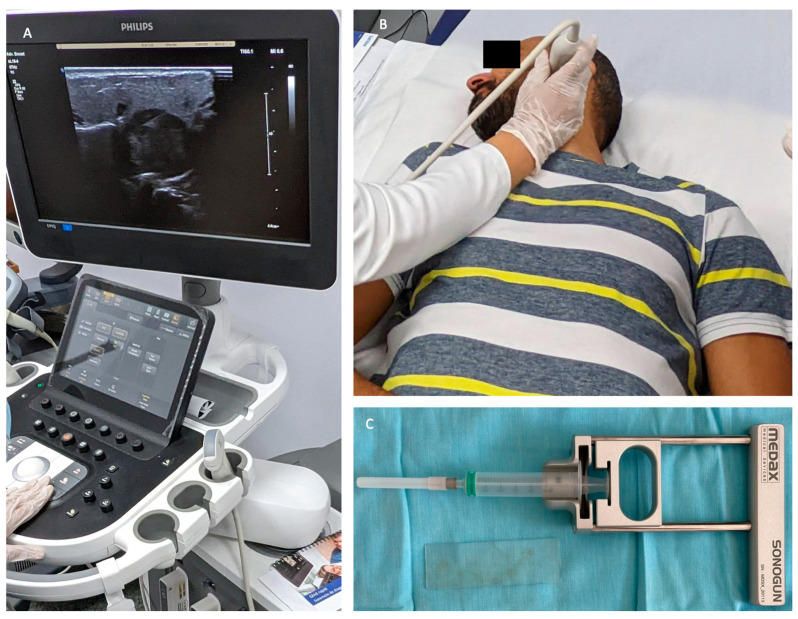
Ultrasound-guided FNAB for a left parotid tumor. Identification of the most representative tumor region for performing FNAB and ultrasound confirmation of the presence of the needle in the tumor (**A**,**B**). The gun-type device used to perform the puncture (**C**).

Employing the FNAB technique under ultrasound guidance increases the diagnostic performance of the procedure because the US confirms the presence of the needle in the most representative area of the tumor and, at the same time, avoids the collection of liquid or necrotic areas from the tumor, which can lead to inconclusive cytological results. Moreover, ultrasound guidance avoids damaging vascular structures during the procedure (Figure 8).

In the present study, five tumors were misdiagnosed when using FNAB (14.2%):

- Two pleomorphic adenomas were confused with Warthin tumors;
- One mucoepidermoid carcinoma;
- One mucous retention cyst was misdiagnosed as a Warthin tumor;
- One Warthin tumor was confused with pleomorphic adenoma.

These incorrect diagnoses may have occurred due to the FNAB samples lacking typical characteristics and containing atypical cells [39]. In addition, mucoepidermoid carcinoma is one of the most challenging tumors to cytologically diagnose [38]. In their study, Lanišnik et al. (2021) reported four malignant tumors that were misdiagnosed as Warthin tumors (4%) from a group of 382 patients who underwent FNAB [31].

Four of the five tumors misdiagnosed when using FNAB were small tumors, measuring between 13 and 28 mm. This may explain the difficulty in obtaining sufficient material for cytological diagnostic interpretation. The diagnostic confusion between mucoepidermoid carcinoma and Warthin tumor following FNAB may be due to the small tumor size (13 mm), which prevented the collection of sufficient and representative material during aspiration, leading to reduced cellularity in the sample and lack of mucin-secreting cells.

The diagnostic confusion of the two Warthin tumors with pleomorphic adenomas can be attributed to the reduced size of the tumors (25 mm and 28 mm, respectively) and their increased mobility, which can lead to difficulties with the FNAB technique. Therefore, the aspirated material was quantitatively reduced, containing moderate cellularity, without cellular atypia, but without identifying lymphocytes in the sample, a characteristic cytologic aspect for Warthin tumor diagnosis.

The confusion of a Warthin tumor with a mucous retention cyst can be attributed to the cytomorphological overlaps of the two tumor entities, and the confusion of pleomorphic adenoma with a Warthin tumor can be attributed to inadequate sampling and/or interpretation error due to the lack of characteristic cytomorphology.

The encouraging results obtained in this study related to the high diagnostic performance of the combined ultrasound and cytological examination (FNAB) in the differential diagnosis of parotid tumors have a significant impact on current clinical practice. Therefore, these two investigations, widely accessible and simple to carry out in an outpatient setting and minimally invasive and non-irradiating, can contribute to the establishment of a precise preoperative diagnosis of parotid tumors, thus avoiding more complex, expensive, and time-consuming imaging investigations, at least in the case of benign parotid tumors located in the superficial lobe.

However, magnetic resonance imaging remains the golden standard imaging investigation for diagnosing parotid tumors and must be performed in all instances where ultrasound fails to provide an accurate diagnosis or raises suspicion of malignancy, in cases where the tumor extends into the deep parotid lobe, or in cases in which the FNAB result is indeterminate or non-diagnostic [9,40].

Moreover, in cases of very rare parotid tumors like solitary fibrous tumors, a single investigation like FNAB is not enough to establish the diagnosis. In these cases, aditional imaging investigations such as MRI are mandatory [41].

We emphasize that certain salivary gland pathologies can manifest through variations in the appearance of the salivary gland papilla. Therefore, its clinical and imaging aspect evaluation should not be overlooked [42].

In addition to these modern imaging and cytological investigations that help establish a correct diagnosis, the choice of surgical technique and the use of modern operating equipment that allows for minimally invasive surgery ensure minimal morbidity for the patient [43].

A useful pathway in the clinical practice for the diagnosis and evaluation of parotid tumors will be suggested. US-guided FNAB and US are the recommended investigations for the initial evaluation of tumors located in the superficial lobe of the parotid gland. These widely available investigations can provide all the information needed to plan the therapeutic strategy if their limitations are considered. In these cases, MRI should be reserved as a confirmatory imaging tool when US and FNAB are unable to provide all the details needed for the preoperative evaluation of parotid tumors.

MRI becomes mandatory for the evaluation of deep lobe parotid tumors and malignant tumors or when FNAB provides an inconclusive diagnosis.

The present study had some limitations, including a single-center design, a limited number of participants in the study, and an uneven distribution of benign and malignant parotid tumors. For additional confirmation of our results, and to reduce the random variation within the results, a large, multicenter study in which evaluation of US, MRI images, and FNAB specimens are performed by multiple radiologists and pathologists, along with an assessment of interobserver reliability, is needed. Interobserver variability would increase the power of the results considering that US and MRI are operator-dependent imaging investigations. A cost-effectiveness analysis of these three diagnostic methods was not conducted in the present study, but it could be of interest for future research.

## 5. Conclusions

Preoperative diagnosis of parotid tumors can be challenging, and it significantly impacts the decision of the surgical treatment strategy.

In the preoperative differential diagnosis of parotid tumors restricted to the level of the superficial parotid lobe, ultrasound, and ultrasound-guided FNAB demonstrated high diagnostic performance.

In this study, MRI and US registered the same diagnostic performance.

Compared with US and MRI, FNAB showed higher overall performance in distinguishing between benign and malignant parotid tumors and in detecting pleomorphic adenomas.

In selected cases of superficial parotid lobe tumors in which the preoperative US diagnosis coincides with the FNAB report, additional cross-sectional imaging investigations are optional to determine the surgical therapeutic strategy. In these cases, MRI may be considered as a confirmatory investigation.

When the diagnosis of parotid tumors using US and FNAB is inconclusive, indeterminate, or non-diagnostic, or when the diagnoses of the two investigations do not coincide, or in case of suspicion of malignancy or extension in the deep lobe of the parotid tumors, MRI becomes mandatory.

## Figures and Tables

**Table 1:** Patient demographic data and parotid tumor characteristics.

Histopathology:	Benign (n = 30)	Malignant (n = 5)	*p*-Value
Age (years) mean value, SD	52 (12.82)	68 (2.86)	<0.001
Gender (female), n (%)	20 (67)	2 (40)	0.3
Living environment (urban vs. rural), n (%)	26 (87)	2 (40)	0.044
Tumor localization (left vs. right), n (%)	16 (53)	1 (20)	0.3
Tumor site within the parotid gland (superficial lobe vs. deep lobe), n (%)	26 (87)	1 (20)	0.006

SD, standard deviation; n, number of patients; %, percent.

**Table 2:** Histopathologic subtypes.

Parotid Tumors	Benign (n = 30)	Malignant (n = 5)
Histopathologic subtype n (%)	Warthin tumor: 16 (53) Pleomorphic adenoma: 12 (40) Basal cell adenoma: 1 (3) Mucous retention cyst: 1 (3)	Acinic cell carcinoma: 1 (20) Salivary duct carcinoma: 1 (20) Carcinoma ex pleomorphic adenoma: 1 (20) Mucoepidermoid carcinoma: 1 (20) Undifferentiated sarcoma: 1 (20)

**Table 3:** Comparative diagnostic performance of US, MRI, and FNAB in differentiating benign from malignant parotid tumors.

Diagnostic Performance	Ultrasound (95% CI)	MRI (95% CI)	FNAB (95% CI)
Sensitivity	80% (28.36–99.49)	80% (28.36–99.49)	100% (39.76–100)
Specificity	97% (82.78–99.92)	97% (82.78–99.92)	97% (83.3–99.92)
Accuracy	94% (80.84–99.3)	94% (80.84–99.3)	97% (85.08–99.93)
Youden Index	0.77 (0.11–0.99)	0.77 (0.11–0.99)	0.97 (0.23–1)
Positive likelihood ratio	24 (3.33–173.17)	24 (3.33–173.17)	31 (4.51–213.17)
Negative likelihood ratio	0.21 (0.04–1.2)	0.21 (0.04–1.2)	0 (0–0)
Positive predictive value	80% (28.36–99.49)	80% (28.36–99.49)	80% (28.36–99.49)
Negative predictive value	97% (82.78–99.92).	97% (82.78–99.92).	100% (88.43–100)
Number needed to diagnose	1.3 (1.01–8.98)	1.3 (1.01–8.98)	1.03 (1–4.34).

CI, confidence interval.

**Table 4:** Diagnostic performance in detecting pleomorphic adenoma—comparison of US, MRI, and FNAB.

Diagnostic Performance	Ultrasound (95% CI)	MRI (95% CI)	FNAB (95% CI)
Sensitivity	75% (42.81–94.51)	75% (42.81–94.51)	78.57% (49.2–95.34)
Specificity	87% (66.41–97.22)	87% (66.41–97.22)	95% (76.18–99.88)
Accuracy	82% (66.35–93.44)	83% (66.35–93.44)	89% (73.26–96.8)
Youden Index	0.62 (0.09–0.92)	0.62 (0.09–0.92)	0.74 (0.25–0.95)
Positive likelihood ratio	5.75 (1.91–17.35)	5.75 (1.91–17.35)	16.5 (2.39–113.93)
Negative likelihood ratio	0.29 (0.11–0.78)	0.29 (0.11–0.78)	0.23 (0.08–0.62)
Positive predictive value	75% (42.81–94.51)	75% (42.81–94.51)	92% (61.52–99.79)
Negative predictive value	87% (66.41–97.22)	87% (66.41–97.22)	87% (66.41–97.22)
Number needed to diagnose	1.61 (1.09–10.84)	1.61 (1.09–10.84)	1.35 (1.05–3.94)

CI, confidence interval.

**Table 5:** Diagnostic performance in detecting Warthin tumors—comparison of US, MRI, and FNAB.

Diagnostic Performance	Ultrasound (95% CI)	MRI (95% CI)	FNAB (95% CI)
Sensitivity	88% (63.56–98.54)	88% (63.56–98.54)	82% (56.57–96.2)
Specificity	94% (72.71–99.86)	94% (72.71–99.86)	89% (65.29–98.62)
Accuracy	91% (76.94–98.2)	91% (76.94–98.2)	86% (69.74–95.19)
Youden Index	0.83 (0.36–0.98)	0.83 (0.36–0.98)	0.71 (0.22–0.95)
Positive likelihood ratio	15.88 (2.35–107.54)	15.88 (2.35–107.54)	7.41 (1.97–27.89)
Negative likelihood ratio	0.12 (0.03–0.46)	0.12 (0.03–0.46)	0.2 (0.07–0.56)
Positive predictive value	94% (69.77–99.84)	94% (69.77–99.84)	88% (61.65–98.45)
Negative predictive value	89% (66.86–98.7)	89% (66.86–98.7)	84% (60.42–96.62)
Number needed to diagnose	1.21 (1.02–2.76)	1.21 (1.02–2.76)	1.4 (1.05–4.58)

CI, confidence interval.

## Data Availability

Data are available from the corresponding author upon reasonable request.
